# Reactivity of Vanadium Nanoparticles with Oxygen and Tungsten

**DOI:** 10.3390/nano12091471

**Published:** 2022-04-26

**Authors:** Francisco Miguel Morales, Marta Escanciano, María Pilar Yeste, Antonio Jesús Santos

**Affiliations:** 1 IMEYMAT: Institute of Research on Electron Microscopy and Materials, University of Cadiz, Puerto Real, 11003 Cádiz, Spain; marta.escanciano@uca.es (M.E.); pili.yeste@uca.es (M.P.Y.); 2 Department of Materials Science, Metallurgical Engineering and Inorganic Chemistry, Faculty of Sciences, University of Cádiz, Puerto Real, 11003 Cádiz, Spain

**Keywords:** vanadium oxidation, vanadium dioxide synthesis, vanadium dioxide doping, X-ray diffraction, differential scanning calorimetry

## Abstract

A mechanistic study was carried out on the optimal methods of fabrication of products containing higher loads of thermochromic VO_2_(M1) fabricated by thermal treatments of V nanoparticles in air, that, once achieved, are more stable than other commercial products upon natural aging or reiterated reheating. At the best temperatures for single runs, 55% of VO_2_ can be attained by the reactions of a limited number of the species initially formed in a process, that, if not stopped, can degrade the product by solid state reactions of oxidations and reductions without O_2_ consumption. This fact supports the use of two-step treatments at lower temperatures and faster cooling rates that reach 65% of VO_2_; such reactions should, ideally, take place in the 550–625 °C temperature range. The impregnation of V with a tungstate salt is an ideal and simple doping platform that can decrease the energy of activation of the 2-cycle process, allowing higher yields and enthalpies of transformation (71% of VO_2_, 26 J/g) than undoped counterparts or trademarks. A good balance is reached for 1% at. of W, with a reduction in T_c_ of 20 °C not significantly resenting the enthalpy of the reversible metal-to-insulator transition. For higher W amounts, the appearance of tetragonal VO_2_, and W alloyed V_3_O_7_ and V_2_O_5_, decrease the fractions of increasingly and effectively doped M1-VO_2_ achieved till 2% of W, a concentration for which T_c_ attains the stimulating values of 35 °C on heating and 25 °C on cooling.

## 1. Introduction

Vanadium is a transition element with an uncompleted electronic structure and a consequent availability of multiple valences involving a complex chemistry. Upon oxidation of this metal, the products can progress to oxide compounds or solid solutions of numerous compositions, or even into many crystalline varieties of the same polymorph. Among all these species, the stable monoclinic dioxide VO_2_(M1) has received the greatest attention, since it transforms to a tetragonal rutile phase of VO_2_(R) during heating at a certain transition temperature (T_c_), with an implicit reversible metal-to-insulator transition (MIT), that makes it the best candidate for many applications in smart glass [1], switching electronics [2], or heat storage [3].

In a previous work [4], we demonstrated simple, fast, dry, cheap, safe and clean methods to generate products with a high load of thermally stable thermochromic VO_2_ particles. These procedures are different to standard ones (mainly hydrothermal routes) because they involve the controlled heat treatment of pure vanadium nanoparticles in air at atmospheric pressure inside an open tube. After a complete design of experiments, and relying on fine control of the operating conditions, it was concluded that the simplest optimum transformations of V into VO_2_(M1) consisted of one cycle of fast heating, followed by keeping temperature fixed in the range of 700 to 750 °C for 400–600 s, and then a further cooling at 0.05 °C s^−1^. On the other hand, by subjecting the V to two consecutive cycles of temperatures and times (625 °C for 5 min) with similar preheating (42 °C s^−1^), but much faster air quenching (~8 °C s^−1^), the best conversion was achieved. Likewise, the best one-cycle pathway was proved to be compatible with VO_2_ doping by the pre-impregnation of V with diluted tungsten salt. Furthermore, we have also shown that analogous methods can be applied to V nano-porous or compact films in order to get M1-VO_2_ layers [5].

The present study complements our previous work, since it delves into the thermodynamics and kinetics behind these kinds of treatments to better understand the mechanisms involved and the reactions of oxidation, as well as, surprisingly, those of reduction. This is done through a comprehensive structural, compositional, calorimetric, thermogravimetric, and mass-spectroscopy characterization panoply applied to crystalline powder mixes formed during, and after, different reaction stages. In addition, a broad study on the effect of the amount of tungsten added to V nano-powders prior to the application of our optimal two-cycles recipe, served to find the ideal W concentration to attain particles with enthalpies of transformation in the range of undoped materials (similar thermochromic behavior to our best W-free products or pure commercial VO_2_), but with a decrease in the temperature of transformation of about 20 °C, which can be useful for certain applications. Furthermore, at higher W concentrations, some mixes with lower performance (poorer in their M1-VO_2_ proportions) reached remarkable T_c_ < 35 °C, which is a proper target for smart windows. This opens the horizon to keep on trying other doping strategies in the future, based on the same approaches.

## 2. Materials and Methods

The used reagents in this study are pure vanadium particles of 100–200 nm (from Nanografi AS, Jena, Germany, ≥99.95% of V) and (NH_4_)_6_H_2_W_12_O_40_·xH_2_O metatungstate hydrate (Sigma-Aldrich, Merck KGaA, Darmstadt, Germany 99.99% trace metals basis). The V or V+W samples were oxidized (i) in air using our own developed methods in an open tube [4], (ii) by similar fast-heating treatments of temperature-programmed oxidation (TPO) combined with mass spectrometry (MS) in atmospheres of O_2_ (5%)/He flowing at 60 mL/min that allow comparable oxygen partial pressures, or (iii) by slower heating in 60 mL/min O_2_ (5%)/He to perform thermal gravimetric analyses (TGA). For kinetic analyses of oxidation (KAO), the samples were heated in a He atmosphere till they reached a target temperature, and, upon stabilization, a O_2_ (5%)/He flux of 60 mL/min was initiated and the chemisorbed oxygen was tracked (indirectly by the mass weight of the products of reaction) at an isotherm with time. The techniques and equipment for studying reactions and materials were: X-ray diffraction (XRD) in a Cu radiation Panalytical X’Pert PRO MPD (Worcestershire, United Kingdom) for phase identification and at times with Rietveld quantification; differential scanning calorimetry (DSC) on TA Instruments Q20 and Q200 systems (New Castle, DE, USA) at standard conditions of heating and cooling (10 °C min^−1^) between 5 and 105 °C in N_2_ inert gas; TGA operated with 10 °C min^−1^ ramps and KAO performed in a TA Instruments SDT Q600; and TPO-MS carried out in a quartz tube reactor coupled to a Pfeiffer Thermostar GSD301T1 model quadrupole mass spectrometer (Aslar, Germany). Each W-doped sample was prepared by incipient wetness impregnation of vanadium in several steps with an aqueous solution of the tungsten salt, and later dried in an oven at 105 °C for 24 h. The corresponding impregnation-drying cycles were completed until homogeneous mixtures were obtained, with different amounts of W with respect to V. In this work the doped specimens were given a notation of #W/V, associated with the numerical atomic percentage of W (#% at. of W) used.

## 3. Results and Discussion

### 3.1. Vanadium Oxidation

**Figure 1:**
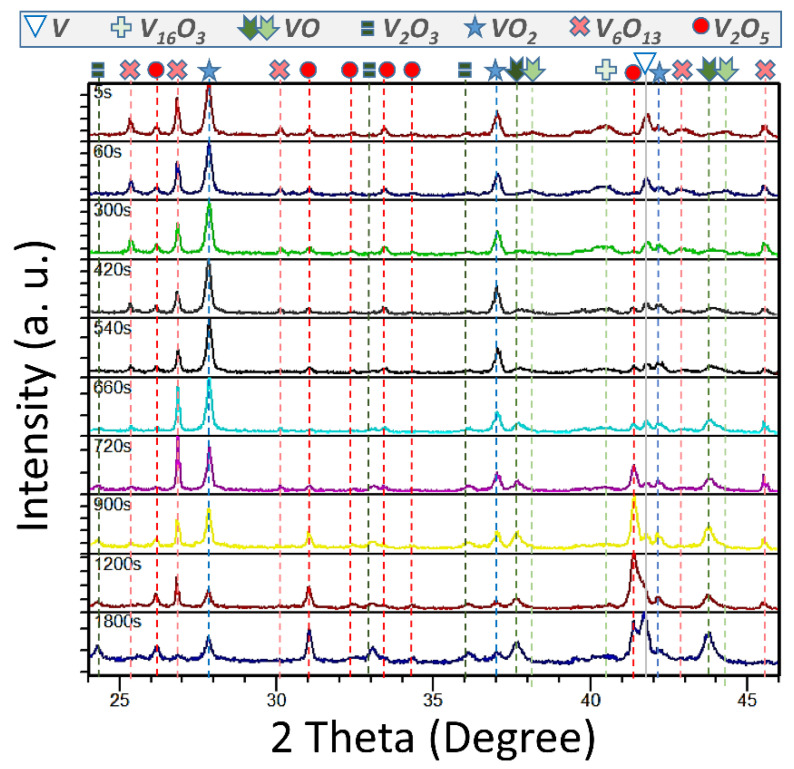
XRD diffractograms for samples obtained after 1-cycle thermal treatments of V nano-powder subjected to 725 °C for different reaction times (labeled in every plot) and fixed heating and cooling rates of 42 °C s^−1^ and 0.05 °C s^−1^, respectively.

**Figure 2:**
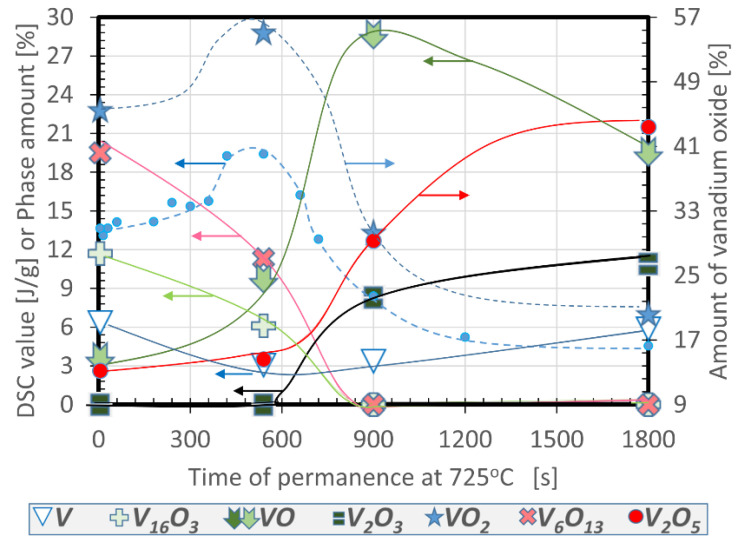
Latent heat (blue dots) and proportions of phases after V oxidations carried out at 725 °C at different times of exposure.

The influence of time at fixed temperature on the V/O system is somewhat complicated as inferred from Figure 1 and Figure 2 for a series of 15 specimens treated in air for 1 cycle at 725 °C at intervals from 5 to 1800 s. Figure 1 presents the XRD identification of phases for 10 of these samples, and Figure 2 shows the quantification by Rietveld refinement of a selection of 4 of them overlapped with the DSC results for all samples.

These tests agree with our previous proposal that expected maximum thermochromic behavior of the product after 6–10 min of reaction at this temperature, and that this property is always linearly proportional to the VO_2_ content. Besides this, it has been observed that the generated crystals are unstable upon calcining, as demonstrated by the absence, or more or less pronounced XRD peaks, due exclusively to the following seven species: V, V_16_O_3_, VO, V_2_O_3_, VO_2_, V_6_O_13_ and V_2_O_5_. There was an absence of the many other alloys or stoichiometric compounds that might be expected to coexist with some of the previous species list, considering the phase diagram for homogeneous mixes [6,7]. However, note that the system does not evolve clearly by accumulating more and more oxidized species at the cost of consuming V and less oxidized ones. On the contrary, the whole mixture seems to saturate in oxygen at a certain concentration, and undergoes solid state reactions within the formed phases, experiencing simultaneous decreases and increases of V oxidation states, without consuming additional gaseous O_2_. These disproportions of intermediate phases through partial reductions and oxidations are noticeable, since, at a particular moment, the oxidative environment plays a less important role than solid system kinetics, thermodynamics, and atom diffusion. In this way, a significant amount of VO_2_ is generated during the first moments of reaction, accompanied by moderate and similar quantities of much less (V_16_O_3_) or more (V_6_O_13_ and V_2_O_5_) oxygen containing crystals, and a small presence of V and VO. When VO_2_ (V^4+^) is at its maximum, V_16_O_3_ and V_6_O_13_ drop, and VO (V^2+^) and V_2_O_5_ (V^5+^) increase. Later on, even when V^4+^ decreases, V_2_O_3_ (V^3+^) suddenly rises, V^2+^ rises to its maximum, and V^5+^ increases at the same time, at the cost of V_16_O_3_ and V_6_O_13_ disappearing. Finally, when the dioxide is at its minimum and balances in quantity with the also decreased monoxide, the pentoxide, trioxide and metal vanadium reach their maximums. It is surprising that reduction reactions let the system partially involute, leading to a slight increase of the metallic vanadium while coexisting with many types of oxides.

Beyond the synthesis mechanisms, we must highlight that these materials have similar thermochromic features, not only being more thermally stable than commercial samples, initially having percentages of M1-VO_2_ over 98%, as was demonstrated in our previous work [4], but also more chemically stable, since after storage of a few grams in air over 9 months we observed a decrease of enthalpy from half to null in commercial samples, and unchanged preservation of this property in our products. In XRD analyses, a clear turn of VO_2_ to slightly more oxidized V_3_O_7_ has been found, which is the reason for the degradation of brand products; a fact described as the reason for the undesirable, but common, oxidation of VO_2_ in other studies [8].

**Figure 3:**
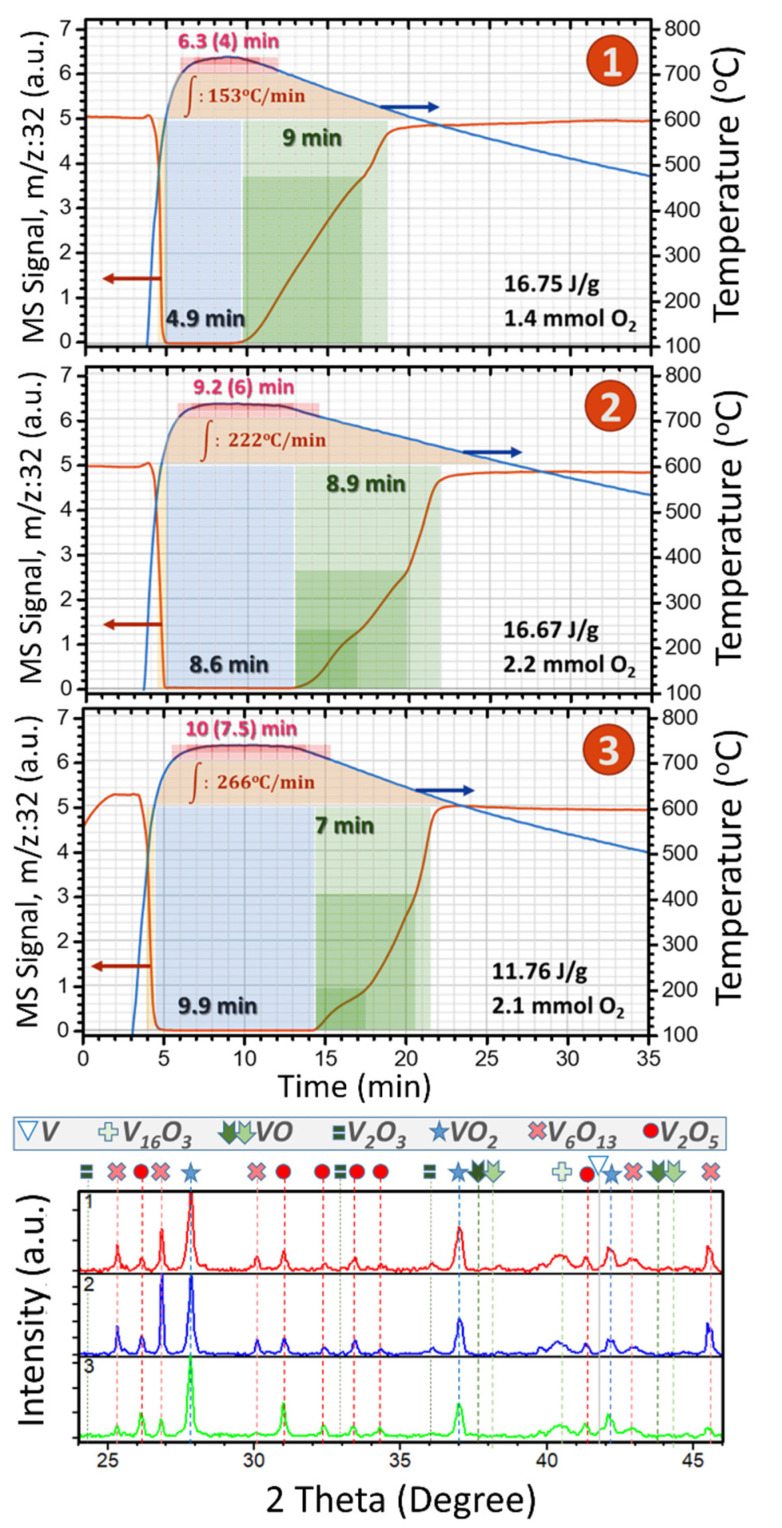
TPO-MS and XRD for V oxidized at 725 °C at different times, with ~10 °C s^−1^ and ~0.05 °C s^−1^ heating and cooling rates, respectively. The total spent of O_2_, and the DSC result for the products are labeled.

TPO-MS experiments give complementary evidence of the reactivity implicated. In similar experiments to those performed in open tube, a flux of O_2_ (5%)/He was circulated through V nanoparticles, and the O_2_ signal (*m*/*z*:32) was registered with respect to temperature and time, and oxygen uptake was calibrated with terbium oxide. Figure 3 presents three cases of TPO-MS from below “1” to more than “2” and to the highest “3” oxidation time as well as the respective X-ray diffractograms of the products normalized to the VO_2_ peaks. The numbers in red on top of the TPO temperature signal (in blue) indicate the time that each sample was subjected to oxidation at 700 °C (in brackets, the time kept between 720 and 730 °C). In addition, the integrated area of the temperatures vs time that the product was above 600 °C is shown in orange.

**Figure 4:**
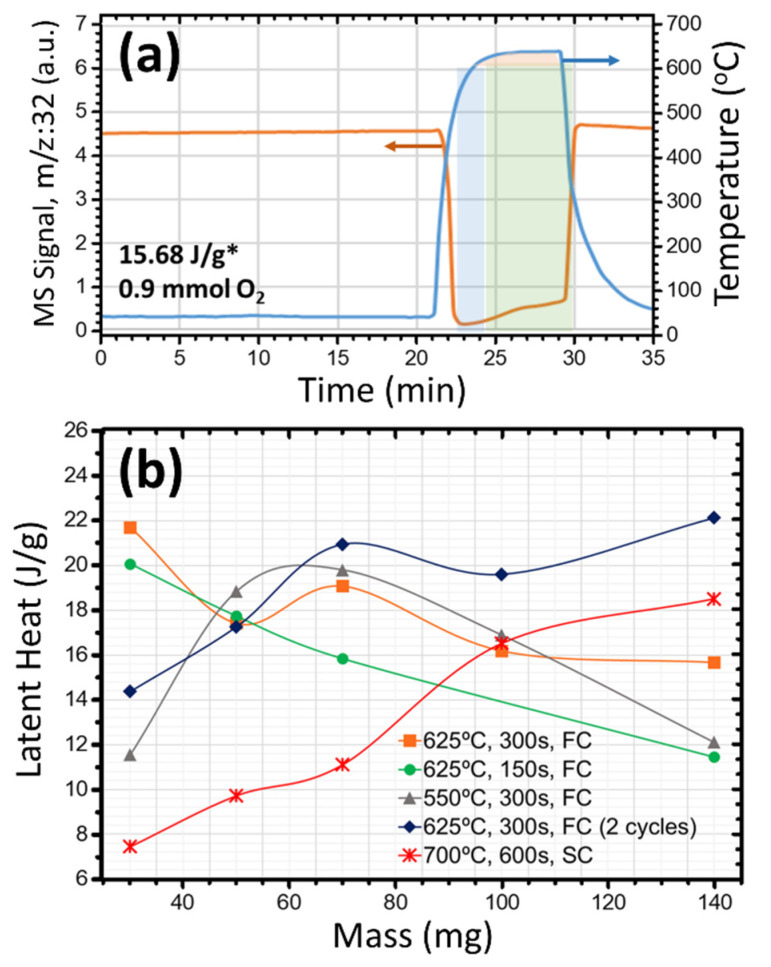
(**a**) TPO-MS for V oxidized at 625 °C for 5 min using ~10 °C s^−1^ and ~1 °C s^−1^ heating and cooling rates, respectively, the total spent O_2_, and the DSC result for a similar product obtained in open tube. (**b**) Value of enthalpy of MIT (metal to insulator) transformation indicative of the VO_2_ yield, for different masses of vanadium subjected to different treatments of oxidation with end of slow (SC) or fast cooling (FC).

In Figure 1 and Figure 2, we observe that at 725 °C the formation of a remarkable amount of VO_2_ is achieved in quite a short time (46% with 5 s of plateau), and that this number could only rise about 9% by increasing the treatment time (55% with 540 s), and this is what made us search for a 2-cycle optimum. This is based on achieving a bit less VO_2_ at 625 °C for 300 s under fast annealing and cooling, and repeating the same process in such a way that the rest of the unreacted metallic V and the slightly oxidized V_16_O_3_ could reach VO_2_ without modifying already coexisting dioxide, allowing a rise of 15%, and, thus, yields of 65% of VO_2_ [4]. The first cycle of this optimum was also tracked by TPO-MS (Figure 4a: 15.68 J/g estimates 50% of VO_2_) although, unfortunately, due to limitations of the setup, the flash annealing could not be replicated to get the TPO-MS behavior of the second cycle. Nevertheless, the plot and decreased oxygen consumption of the first cycle is indicative of less O demanding reactions, promoting a blocking of the full ingestion mode (the horizontal line indicating null O signal is almost avoided, and the total oxidation time is reduced to 7 min). Note that the range of temperature and times used in this plot are the same as those of Figure 3, to allow better comparisons.

One may think that using smaller masses of precursor for reduced temperatures, combined with fast cooling. could have a similar effect to repeating cycles. In this light, trials subjected to smaller portions of V at different temperatures were carried out for the 1- and 2-cycles optima (700 °C/600 s/SC: slow cooling and 2 × (625 °C/300s/FC: fast cooling), respectively) and for 1 run at less reactive temperatures (550–625 °C) and fast cooling. Attending to the DSC values (Figure 4b), it can be seen that the VO_2_ yields of these samples did sometimes improve. See, for example, that 30 mg at 625 °C or 70 mg at 550 °C produce similar yields to the optimum of 2 cycles with 140 mg. Anyway, no operative advantages were found for our reactor, but this information can be used favorably for fast fabrication industrial systems using a continuous flow of a thin bed of V nanoparticles passing through a less energetically demanding furnace equipped with a fast-cooling system on exit.

### 3.2. Vanadium plus Tungsten Oxidation

**Figure 5:**
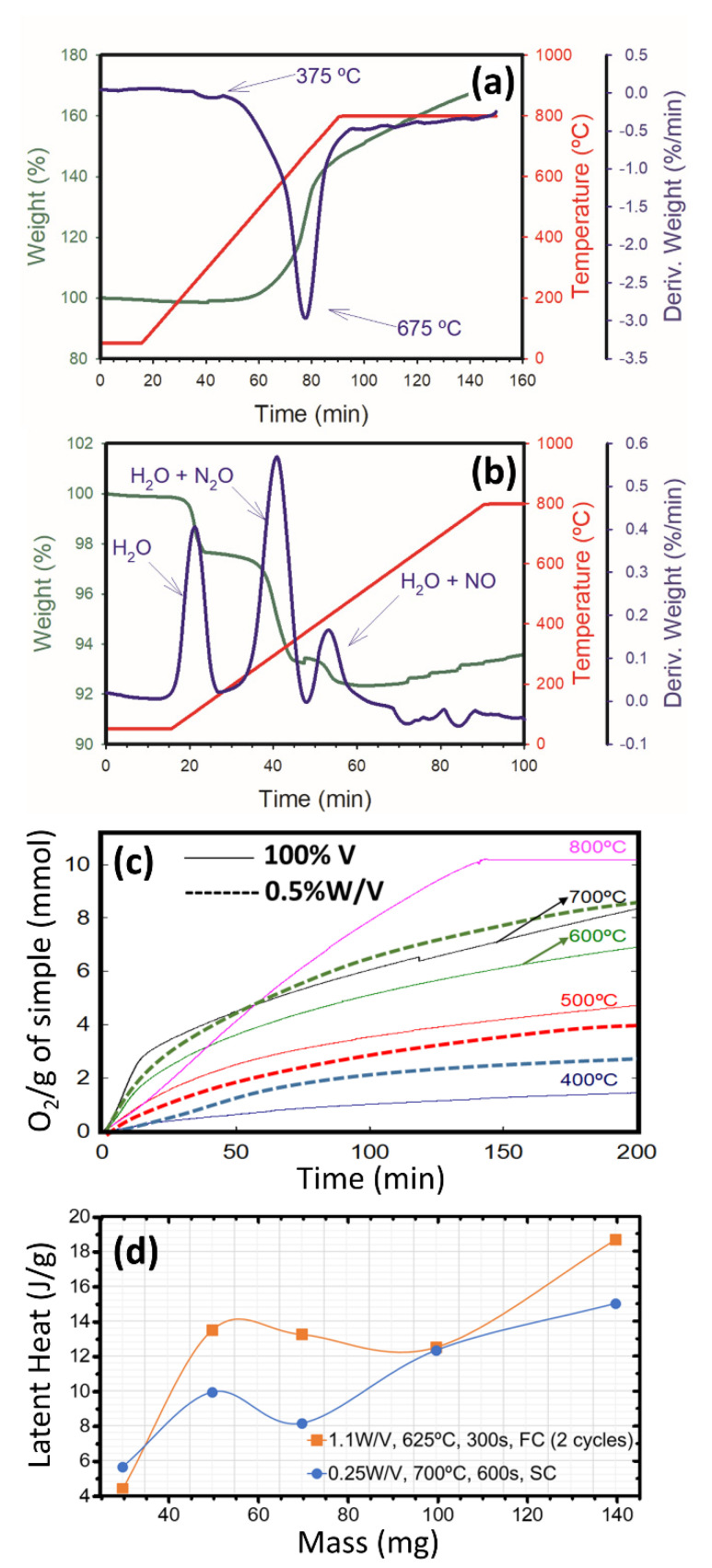
TGAs of V NPs (**a**) and of (NH_4_)_6_H_2_W_12_O_40_·xH_2_ (**b**) in oxidative atmosphere. (**c**) Kinetic curves for isothermal processes of oxidation. (**d**) Values of enthalpy of MIT transformation at heating indicative of the VO_2_ yields, for different masses of V plus W subjected to optimal treatments of oxidation for pure V: 1-cycle with slow cooling (SC), and 2-cycles with fast cooling (FC).

Kinetic studies of the oxidation of V and of V+W were carried out, together with a TGA study of both isolated precursors in oxidative atmosphere, in consideration of the effect of treating different masses of V(+W), or reducing them, before oxidation. Figure 5a,b show the increase or decrease in weight of the vanadium and the tungstate hydrate, respectively (green line). The derivatives versus time plot of these (in blue) highlights the inflection points associated with key moments of absorption/chemisorption or desorption/decomposition: while V starts to oxidize at about 375 °C, showing a maximum at 675 °C and a saturation at near 800 °C. The tungsten salt ((NH_4_)_6_H_2_W_12_O_40_·xH_2_O) disintegrates progressively to form tungsten oxide by dehydrating at three moments, near 200 °C, at about 300 °C in combination with nitrous oxide desorption, and a bit over 400 °C joined to discharging nitric oxide, with no further remarkable loss of weight. On the contrary, there was some stepwise oxidation, which indicates expected [9] and desired complete decomposition at temperatures inferior to those used for thermal treatments of vanadium in open tube. Note also that by mass spectrometry of V in oxidative atmosphere and progressive heating, we were able to detect very slight signals associated with the evaporation of water and carbonates from the surfaces of the nanoparticles at about 500 °C (not shown here). Moreover, analyzing the data of chemisorbed O_2_ (Figure 5c) for V or 0.5 W/V, the changes of slope in the curves evidence the real velocities at which the process of oxidation happens isothermally. The point of equilibrium for the quantity of chemisorbed O_2_ occurs when the sample is at its maximum absorption capacity. For pure V, this value was set to 1.44, 4.72, 6.91, 8.34 and 10.18 mmol of O_2_ per gram of sample, at 400, 500, 600, 700 and 800 °C, respectively. By inputting these constants in pseudo first order and second order rate equations, considering two simplified kinetic models [10], the best fits were found for a first order kinetic having R^2^ > 0.996 in all cases, while for a second order kinetic the correlation coefficients deviated from the unit in a more noticeable way. This suggests that the energy of activation for vanadium oxidation, considering the Arrhenius equation, is 14.25 kJ/mol. It is noticeable that W modifies the intake of O_2_ at each temperature, and, assuming that, for W impregnated V, the kinetics is also of first order, considering the data associated to the three dashed plots, an energy of activation of 21.20 kJ/mol was calculated. The general indication is that promoting oxidation in doped systems is energetically more demanding.

Concerning the optimization of VO_2_ doping using air as the oxidation source and the considered nominal V mass (140 mg) for the pre-impregnation of W on V before the subsequent optimal 1-cycle treatment in open tube, the measured endothermic latent heats for the products of 0.05, 0.1, 0.25, 0.5, 1 and 3% of W were 14, 10, 15, 11, 8 and 3 J/g with a T_c_ at heating of 66, 64, 60, 61, 63 and 67 °C, respectively. In this way, a reduction treatment of 24 h in H_2_ atmosphere at 900 °C was applied after tungstate impregnation to get rid of possible partial oxidation and promote desorption of molecules remaining from these handlings. For 0.5 W/V, additional reductions of 12 and 48 h were carried out. Nevertheless, after thermal treatment at 700 °C for 600 s, there was only ~1 J/g increase in enthalpies of transformations, at the cost of ~1 °C increase in the T_c_, with respect to their unreduced counterparts, so the effect of reduction was not relevant at all. In the optimal 2-cycle scenarios applied to W containing powders, the reduction pretreatment was also not advantageous, with a similar modest effect for thermochromic variations. The influence of diminishing masses in the open tube treatment was also considered in the case of some V:W samples (Figure 5d). For the optimum of one run applied to 0.25 W/V, or the optima of 2-runs to 1.1 W/V, there is an evident and disadvantageous yield decay.

**Figure 6:**
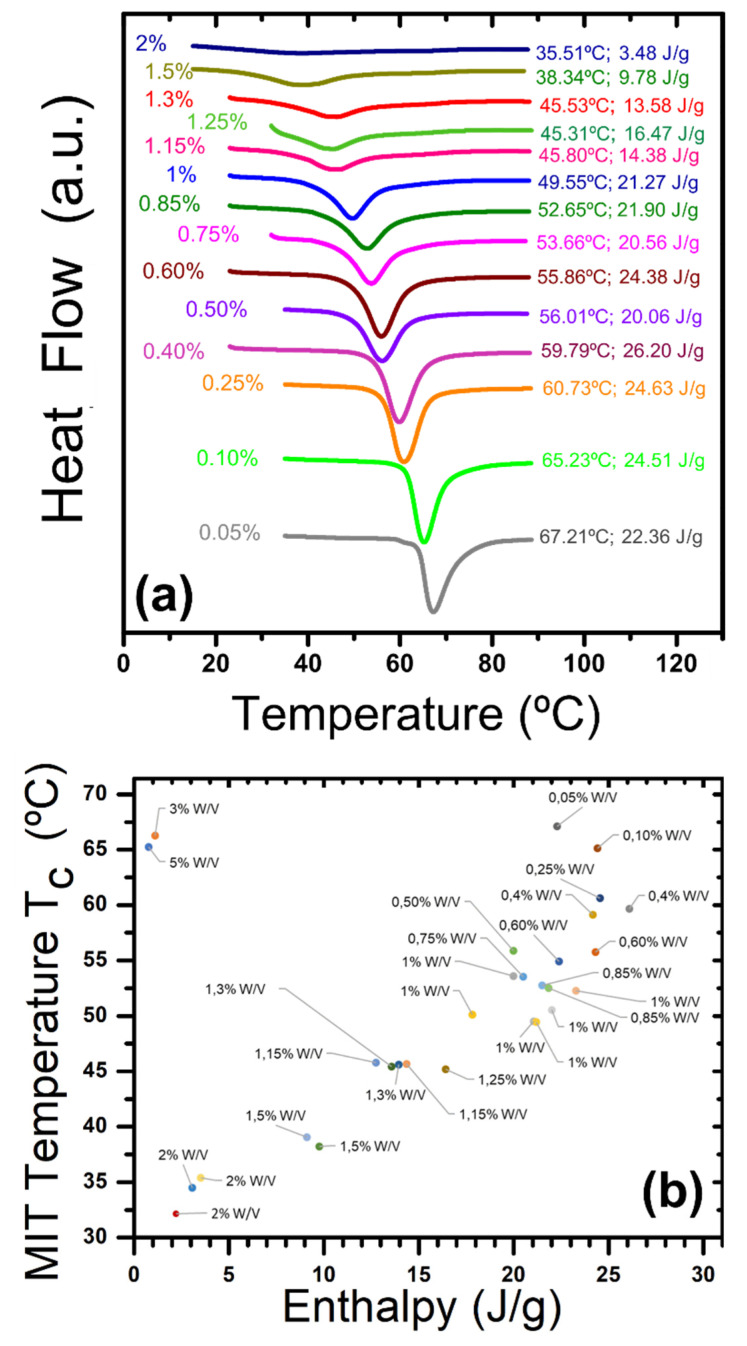
(**a**) DSC endothermic peaks for most of the tested compositions. (**b**) Tc vs. ΔH for all W doping trials of 2 cycles.

Even though the achieved effect by doping is very modest in the one cycle optimum, there are significant results for the two cycles treatments combined with W pre-impregnations. Figure 6 shows the effect of continuously increasing the content of tungsten on the position, shape and area of the DSC endothermic peak related to the heating of the product containing effectively doped portions of vanadium dioxide. Note that every exothermic peak at cooling was always relatively symmetric to its couple, and positioned at a randomly lesser temperature between 5 and 8 °C lower, so this value of the hysteresis gap does not follow any tendency. Figure 6a presents one example of the DSC experiment for most of the tested compositions, showing a clear monotonic diminishing of T_c_ and ΔH with W concentration. Figure 6b compiles these properties for the same samples, as well as other repetitions or different concentrations.

**Figure 7:**
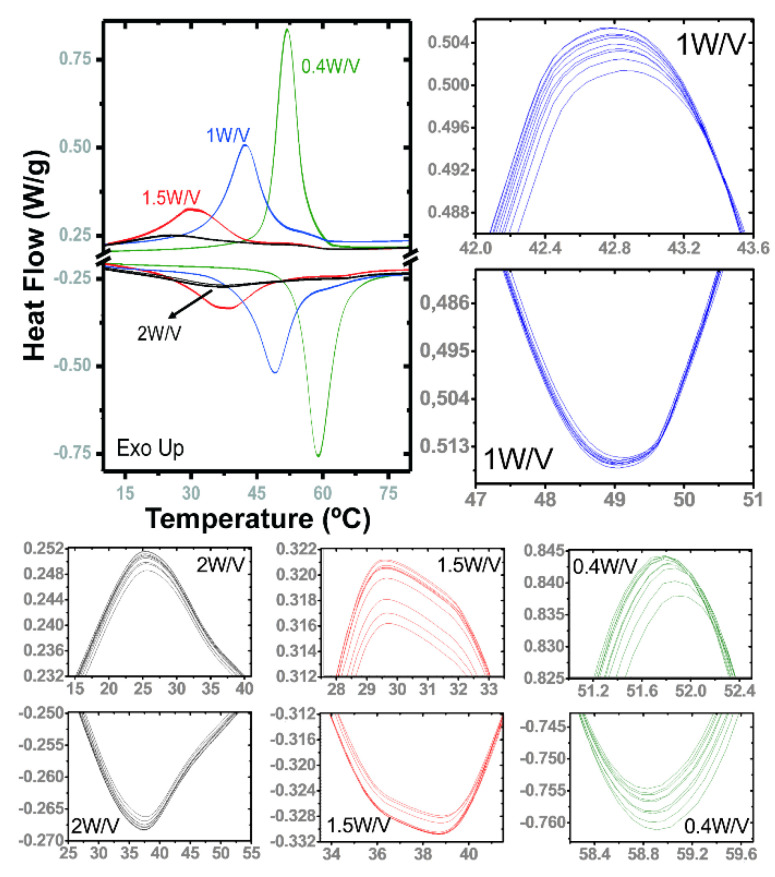
DSC plots of 10 cycles of heating and cooling applied to some representative samples of W/V tested compositions, with the detail of the top of the peaks in the same range of heat flow for comparison, demonstrating a high level of replication.

It is remarkable to mention that some specimens showed enthalpy values even higher than the undoped VO_2_ achieved by this method (T_c_ = 60 °C, ΔH = 26.2 J/g), as one 0.4 W/V sample with a 4 J/g increase and a T_c_ that is 8 °C lower. The general trend looks to break for samples with a high content of W (3 and 5%), but the 2% samples were able to reach small portions of effectively doped dioxide with outstanding T_c_ of 32–35 °C heating; that is an ideal value for their implementation in smart windows. Probably the best samples, considering balance of thermochromic performance, are those of 1 W/V, that reached a decrease of about 20 °C in T_c_ without significant sacrifice in latent heat with respect to the pure VO_2_ synthesized by the same method, or commercial M1-VO_2_. Another important issue is good stability upon continuous heating and cooling. Figure 7 presents the DSC plots for 10 thermal cycles applied to 4 representative samples, with peaks zoomed in the same narrow range of heat flow, demonstrating recurrent intermittences for all the cases without apparent degradation. Note that the repetition is especially accurate in the case of 1 W/V heating, and all the exothermic peaks are very symmetric and appear at lower temperatures. The peak at cooling for sample 2 W/V stands out, which is interestingly centered close to 25 °C. Nevertheless, as the sample contains more tungsten, the peaks distribute their areas in a wider range of temperature, that can have advantages for heat storage, but not for fast switching.

**Figure 8:**
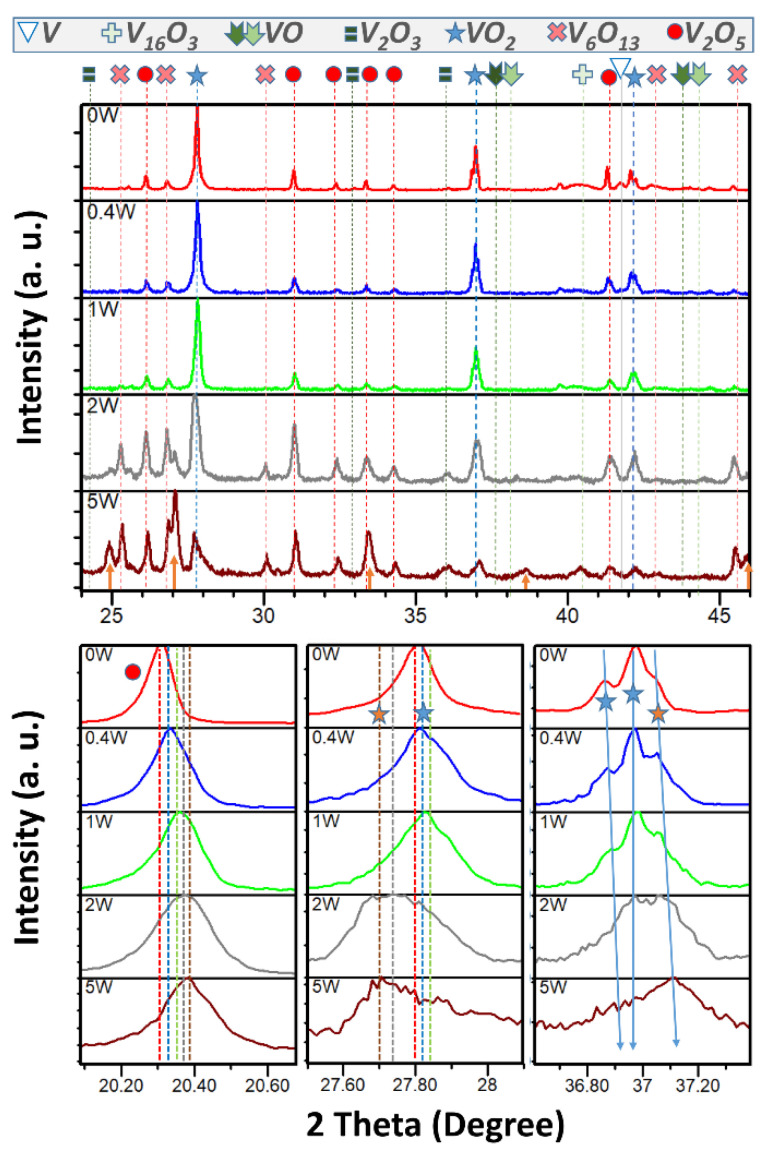
X-ray diffraction patterns for samples heat treated with the optimum 2-cycles method, together with some insets of amplifications. Note that arrows and stars in orange are in diffraction positions proper to the (V_0.8_W_0.2_)_3_O_7_ and R-VO_2_ phases, respectively.

Observation of the XRD plots of the doped samples (Figure 8), allows some associations to previous results to be made. For example, the relative percentage of V_2_O_5_ in this series was quantified as 30.2, 28.5, 28.3, 45.7 and 33.1%. In the often-selected range, the diffraction peaks assigned to pentoxide in a reference undoped specimen fabricated by the same optimum 2-cycles method (0 W/V) are at 26.1, 31.0, 33.3, 41.3, and 34.3°, and they look to move more and more to the right in the rest of the samples as more tungsten was used. This is more visible for the zoom of the main diffraction peak of the orthorhombic V_2_O_5_, at 20.3°, that consecutively shifted towards higher angles when increasing W content, indicating the formation of a solid solution (W/WO_3_ successfully inserted and highly dispersed into the V_2_O_5_ framework) accompanied by a progressive diminution of the unit cell. Although the dopant cation has a larger atomic radius (W = 142 pm) than the host cation (V = 135 pm), the dopant is trivalent (W^3+^) and the host is pentavalent (V^5+^). Consequently, the reduction in the lattice constant of the host material was explained [11] as being caused by a deficiency of dopant ions accumulating in the vacancy positions of the oxygen ions in the pentavalent host lattice.

This could also be the reason for the shrinking of the M1-VO_2_ lattice from 0 W/V to 0.4 W/V and then to 1 W/V, since the host is a quadrivalent cation (V^4+^). For the emphasized peaks at about 27.8, 36.8 and 36.9° this is proven (blue star peaks in the zoomed regions at the bottom of Figure 8). However, diffraction of samples 2 W/V and 5 V/W seem to break this trend, and their VO_2_ signals (red star) also fit with the presence of the tetragonal phase (ICDD database code 04-008-7669) rather than with monoclinic dioxide. In 5 W/V this R-VO2 is 7.8% and the M1 phase was present in a similar small percentage and undoped, as seen in Figure 6b (a negligible associated signal with T_c_ proper of pure M1-VO_2_ is present). This ineffective doping of the M1 phase must also be the situation regarding 3 W/V, for which XRD analyses were not performed, but evident in the DSC result. The tetragonal phase of the dioxide is also identified to coexist with a moderate quantity of doped M1 phase in 2 W/V (11.9% of tetragonal and 23.7% of monoclinic), the diffraction peaks of which are partially eclipsed by the tetragonal ones at certain positions. Although this is not a high yield for monoclinic dioxide, the idea of its effective doping is supported by the observed DSC endothermic and exothermic peaks’ values and positions shown in Figure 7. Discernment between both M1 and R phases in XRD can be delicate but visible with changing temperatures [12,13,14], sharing theoretical reflections of both pure compounds with similar relative intensities and positions. However, compared to the doped monoclinic peaks, expected at a bit over 27.8° and 36.8°, the cell of the R-VO_2_ reflects near these values at a few less, or a few more, units of decimals of the 2θ angle, respectively. In the low W content samples, the slight presence of tetragonal VO_2_, detected as a shoulder of the monoclinic peak at 36.9°, was found to be insignificant as happens for V_6_O_13_, that was quantified in less than 1% in 0.4 W/V and 1 W/V, while M1-VO_2_ are estimated in 71 and 70%, correspondingly, and mostly the rest can be assigned to the pentoxide. Although the R phase is metastable at ambient temperature, its small difference of formation energy with respect to pure M1-VO_2_ [15], and the W introduction that theoretically decreases this energy difference [16], may explain the coexistence of both phases, a circumstance that has been reported previously for particles of 1 and 2% of W [14]. In VO_2_ films, the onset for the trend to form tetragonal, instead of monoclinic, was found between 0.9 and 2.1% of W [12], which is in good agreement with our studies.

In addition, for the more doped samples, extra XRD peaks at 25, 27 and 46° (yellow arrows) index with an extra tetragonal phase (ICDD 00-025-1007). In sample 5 V/W, the presence of this (V_0.8_W_0.2_)_3_O_7_ compound (14.1%), coupled with V_2_O_5_ (33.1%) and V_6_O_13_ (34.2%), explains why the thermochromic property is ruined, as likely happens for 3 W/V. In sample 2 V/W, (V_0.8_W_0.2_)_3_O_7_ is 2.5%, V_6_O_13_ is 16.2%, V_2_O_5_ is 45.7%, and the mixes of VO_2_ sum 35.6%. In this way, the trend of vanadium to oxidize seems to be amplified by the presence of tungsten, and this is contrary to our previous hypothesis based on kinetics studies for a 0.4% of W, so, there must be a concentration limit when this tendency reverses, and probably this is over 1% W, when the enthalpies of transformation of the products begin to be smaller than those of the undoped one.

Comparing with other studies dealing with W alloying, and seeking the most effective dopant for lowering M1 to R transition temperature, and a recent literature survey on the formation, stability, and thermochromic values of V_1-x_W_x_O_2_, it is accepted that tungsten often decreases T_c_ by approximately 20–26 °C per at. % at the expense of degrading its thermochromic performance [14]. This is in agreement with our observations, regardless of the fact that most of the articles checked obey the valuable quantitative yield information based on DSC enthalpies or XRD percentages of monoclinic VO_2_ as made in the present work, and focus only on the variations of T_c_. An example of this can be found in a study that shows DSC curves of VO_2_ powders with W-doping of 0.71%, 1.32%, and 1.58%, with apparent loss of latent heat numerically not claimed and variations of the T_c_ less efficient than in our work: from 69.5 °C (undoped) to 56.2, 43.9 and 37.8 °C, respectively [17]. Other studies demonstrate similar decays in enthalpy to ours but also with less effective decreases of T_c_ with W content. For instance, for 1 and 1.5% the heating and cooling transitions were 48/42 °C and 40/34 °C, respectively [18]. In another study, for undoped VO_2_ of 23.50 J/g, similar variations in the endothermic enthalpies along MIT were found, declining respectively to 16.79, 3.62 and 2.01 J/g when W doping grows at 0.5, 1.0 and 1.5 at.%, which is also accompanied by a decrease in their critical temperatures (67 to 57, 43 and 31 °C) and hysteresis widths (10 to 9, 8 and 5 °C) [19]. To the best of our knowledge, only a few works dealing with doped VO_2_ particles demonstrated that doping can be performed without a small loss of enthalpy, probably based on the small size of the particles [20,21].

## 4. Conclusions

A mechanistic study was carried out on the optimal methods of fabrication of products containing higher loads of thermochromic VO_2_(M1), achieved by thermal treatments of V nanoparticles in air. At the best temperatures for single runs, about 46/30/10% of M1-VO_2_, V_6_O_13_ and V_2_O_5_, respectively, combined with V_16_O_3_ and VO were formed in a few seconds at the cost of consuming most of the V. In the following minutes, a maximum of 55% of VO_2_ was attained by the reactions of species initially formed, that compete at the same time for the oxygen of the environment and for that contained in themselves. Upon M1-VO_2_ saturation, the system evolved without O_2_ consumption, even though in open tube, and during subsequent solid-state reactions, including oxidations and reductions, VO_2_ decreased, VO and V_2_O_5_ increased, V_2_O_3_ rose, and V_16_O_3_ and V_6_O_13_ disappeared. In the whole process, V stayed at a similar level, even slightly increasing with time, which is remarkable for an oxidative ambient. All this knowledge served to propose two stages treatment at lower temperatures and faster cooling, that force the system to enlarge the processes that occur in the first seconds at higher temperatures, when V, V_16_O_3_ are the precursors to form M1-VO_2_ but the smaller temperature is not enough to promote dioxide oxidation to a high extent, yields being allowed till about 65% of VO_2_. The products were demonstrated to be very stable with time, compared to commercial reagents, and no advantages were found when the masses treated thermically were decreased. Thermo-gravimetric studies showed that the low-temperature extent of more controlled V oxidation kept increasing from 375 to 675 °C, supporting previous results that established the 550–625 °C range as the best compromise to have relatively fast processes. Moreover, the impregnation of V powders with meta-tungstate hydrate appeared to be an ideal precursor of the 2-cycles method to achieve effective dioxide doping for lowering T_c_, and TGA and kinetics studies indicate that more than 400 °C is needed to decompose the W salts. The studies also show that the energy of activation for oxidizing the bare, or slightly W (0.5%) impregnated, vanadium is 14.25 and 21.20 J/mol, respectively. This may explain the fact that, for these concentrations, the dioxide yields, as well as the associated thermochromic behaviors, outperform those of the undoped product (0.4 W/V showed 71% of VO_2_ with ΔH > 26 J/g). A good balance is achieved with 1% at. of W, with similar enthalpies of transformation than for the undoped one, but an interesting reduction in the T_c_ of about 20 °C. At higher doping levels, the appearance of the metastable tetragonal dioxide and of doped compounds, based on V_3_O_7_ and V_2_O_5_, ruin thermochromic characteristics. Although decreasing fractions of M1-VO_2_ with increasing doping obtained till 2% of W, demonstrate the remarkable fact that T_c_ temperatures of 35 °C on heating, and of 25 °C on cooling, offer an interesting range for smart windows applications, and deserves further studies in the future.

## Data Availability

UCA institutional repository https://rodin.uca.es/handle/10498/6086.
